# Compliance with safety measures and risk of COVID-19 transmission among healthcare workers

**DOI:** 10.2144/fsoa-2021-0094

**Published:** 2021-10-29

**Authors:** Nawaf J Shatnawi, Zaid Mesmar, Gaith A Al-Omari, Wesam AL-Sheyab, Nabil A AlZoubi, Mohammed AL-Ghazo, Shadi Hamouri, Ibraheem AL-Faori, Ali Bani-Essa, Ismail Matalka, Yousef S Khader, Anwar Batieha

**Affiliations:** ^1^Department of General Surgery, Faculty of Medicine, Jordan University of Science & Technology, Irbid, Jordan; ^2^Department of Pathology & Laboratory Medicine, Faculty of Medicine, Jordan University of Science & Technology, Irbid, Jordan; ^3^Department of Nursing, Faculty of Nursing, Jordan University of Science & Technology, Irbid, Jordan; ^4^Department of Public Health & Biostatics, Faculty of Medicine, Jordan University of Science & Technology, Irbid, Jordan

**Keywords:** compliance of HCWs, COVID-19, healthcare workers, King Abdullah University Hospital, SARS-CoV-2, total anti-SARS-CoV-2 immunoglobulin assay

## Abstract

**Aim:** This study aimed to determine the compliance of healthcare workers (HCWs) with the hospital safety measures and the prevalence of hospital-acquired COVID-19 infection among them. **Methodology:** HCWs at King Abdullah University Hospital (KAUH) assigned for COVID-19 patients between 18 March and 10 June 2020 were tested for past infection using total anti-SARS-CoV-2 immunoglobulin assay, demographic data and compliance with safety measures were assessed using a questionnaire. **Results:** A total of 340 HCWs participated in the study, 260 were close direct care. Three HCWs tested positive for total anti-SARS-CoV-2 immunoglobulin. Close direct care were more compliant with personal protective guidelines than those providing direct care. **Conclusion:** HCWs compliance with personal protective guidelines might explain the low prevalence of COVID-19 infection in hospital settings.

## Lay abstract

Hospitals around the globe have implemented safety measures in order to decrease the risk of spreading the virus among healthcare workers (HCWs); our aim in this study is to assess the compliance of HCWs to the safety measures introduced in our hospital and the prevalence of contracting COVID-19 among them using total anti-SARS-CoV-2 immunoglobulin assay. A total of 113 physicians and 227 nurses participated in the study; results showed a high level of compliance among HCWs working in close direct care and a relatively lower level of compliance among those providing direct care. Three of the HCWs tested positive for the total immunoglobulin assay, indicating the importance of adhering to the safety measures to decrease the risk of contracting the virus.

The novel SARS-CoV-2 pandemic was first reported in 2019 in Wuhan, China. The infection spread rapidly to be recognized as a global pandemic on 11 March 2020 [1,2]. Therefore, countries around the globe, including Jordan, imposed unprecedented restrictions and adopted safety measures attempting to limit the spread of the virus among public and healthcare facilities [3–5]. Healthcare workers (HCWs) are exposed to an elevated risk relative to the general population because of closer contact with infected patients especially for those who are designated for taking care of COVID-19 patients [6]. Even some would suggest that its considered as one of the occupational injuries [7]. The availability of personal protective equipment (PPE) in addition to the level of HCWs awareness/compliance with personal protective guidelines is crucial in reducing the rate of the viral transmission especially with the redeployment of less experienced clinical staff to frontline positions [8,9]. Studies showed that standardized and strict adherence to PPE can reduce nosocomial transmissions [10]. Screening for hospital-acquired SARS-CoV-2 infection is vital for limiting subclinical transmission to noninfected patients, family members and the community as a whole [11]. Reverse transcription PCR (RT-PCR) assay for nasopharyngeal swabs is the diagnostic test for active infection [12]. However, the timing of the RT-PCR assay is a crucial limitation in the molecular diagnostic perspective, where false-negative results may ensue, especially at the early incubation period and late recovery stages of infection [13,14]. Therefore, RT-PCR is limited to confirmation of active disease and contact tracing [13,14]. On the other hand, the usage of the early developed rapid antigen tests has shown promising results in detecting COVID-19 among HCWs due to its short term of detection but it had less sensitivity compared with the RT-PCR tests [15].

Consequently, screening by anti-SARSCoV-2 immunoglobulin assay can be more informative in detecting past asymptomatic infections and possible HCWs acquired immunity [16]. King Abdullah University Hospital (KAUH) is a tertiary teaching institution designated to treat confirmed COVID-19 patients in northern Jordan [17,18]. We aimed to determine the compliance of HCWs with hospital safety measures and the prevalence of hospital-acquired SARS-CoV-2 infection among physicians and nurses using anti-SARS-CoV-2 immunoglobulin assay.

## Materials & methods

This is a cross-sectional study carried out at KAUH between 17 March 2020 and 10 June 2020, reflecting the first wave of COVID-19 in the country. During this period, 177 confirmed COVID-19 patients were hospitalized. A total of 344 HCWs (physician and nurses) were assigned to provide direct care for hospitalized COVID-19 patients. These HCWs were screened by RT-PCR assay of nasopharyngeal swap during May 2020 with no positive results [18]. About 4 weeks later, they were asked to participate voluntarily in this study. Physicians and nurses at KAUH who were assigned to taking direct care of hospitalized COVID-19 patients and had at least one RT-PCR assay were included in this study, while the other HCWs and employees were excluded. Close direct care in our study included those who provided care to COVID-19 patients within a distance of ≤1.5 meters and ≥15 min/24 h or participated in aerosol-generating procedures. Any other type of COVID-19 patient’s care in the designated locations was defined as direct care [19,20]. A questionnaire was structured and sent through emails with a covering letter explaining the purpose of the study. Participation required signing a consent form, answering the questionnaire and providing a blood sample for the total anti-SARS-CoV-2 immunoglobulin assay. The questionnaire included demographic data, occupational characteristics, nature of COVID-19 care, work allocation sites, working hours per shift, comorbidities (hypertension, allergies and smoking history), history of flu-like symptoms during COVID-19 care periods (fever, runny nose, cough, generalized weakness, muscles and joints pain, sore throat), annual influenza vaccination, contact with confirmed COVID-19 patients (outside the hospital, family member) and co-worker confirmed by RT-PCR assay, sleeping pattern changes (difficulty going to sleep, interrupted sleep, nightmares) and changes in regular daily activities (appetite, socialization, irritability, concentration). Compliance with personal protective guidelines recommended PPE and the hospital infection control and prevention (ICP) policy were assessed separately with choices of (always/most of the times/frequently/occasionally). Satisfaction with the hospital education/training process for using PPE (highly satisfied/satisfied/satisfied with preservation/not satisfied) was also included. Sleep pattern changes were considered positive if the HCW answered yes with one or more sleep components. Changes in regular daily activity were considered positive if HCW replied yes with one or more of the question components. Compliance with personal protective guidelines, ICP policy and PPE use was defined if participants reported that always/most of the time. Being satisfied with education/training for PPE use was defined if participants’ answers were highly satisfied/almost satisfied. The hospital database and records of the ICP committee were used for relevant data related to the study (date of patient admission and discharge, total admitted confirmed cases, assigned HCWs with their RT-PCR assay, mean hospital stay for confirmed patients).

About 5 ml of blood was collected in a plain tube from each participant starting from 23 June 2020. The sample was centrifuged and stored in deep freeze for assays. Total anti-SARS-CoV-2 immunoglobulin Roche (Basel, Switzerland) kits were used for chemiluminescent immune-assay on participants’ serum samples running on Cobas instrument (Roche). The test was considered positive according to the kit manufacturing company recommendation. The test kit was calibrated in our lab by the kits control provided. This study was approved by the institutional review board of Jordan University of Science and Technology and the hospital authorities (150/132/2020). Statistical analysis was performed using IBM SPSS Version 24. Data were described using means and percentages. Chi-square test was used to compare percentages and independent *t*-test was used to compare two groups means. A p-value of less than 0.05 was considered statistically significant.

## Results

A total number of 340 HCWs participated in the study, 113 (33.2%) physicians and 227 (67.7%) nurses. Of those, 242 (71.2%) were males. The mean standard deviation (SD) age was 31.09 (±5.35) years for males and 30.28 (±4.23) years for females. There were no significant differences related to the studied variables between males and females. Table 1 illustrates the differences between males and females in relation to demographic, occupational, health characteristics, comorbidities, compliance and satisfaction. Table 2 compares physicians and nurses for demographic data, occupational characteristics, health characteristics, compliance with protective guidelines and the recommended PPE and satisfaction with education/training for PPE use. A total of 260 (76.5%) HCWs were providing close-direct care and 80 (23.5%) were providing direct care for hospitalized COVID-19 patients. The close direct care providers were younger, with a shorter period of experience and worked for longer shifts (differences are significant). The prevalence of sleeping pattern changes was 45.8% for the close direct care providers, compared with 30% for direct care providers (p = 0.014). The nature of COVID-19 patient’s care with demographic, occupational, health characteristics are presented in Table 3.

**Table 1.** Females compared with males in relation to; demographic, occupational, compliance with hospital safety measures and satisfaction with education/training for personal protective equipment use.

Compared variables	Female (n = 98)	Male (n = 242)	p-value
Age (years), mean (SD)	30.28 (4.23)	31.09 (5.35)	0.42
Experience (years), mean (SD)	6.09 (4.22)	6,61 (4.86)	0.283
Working shift (hours), mean (SD)	15.36	15.30	0.941
Close direct care	77 (78.6%)	183 (75.6%)	0.672
Smokers	26 (26.5%)	54 (23.3%)	0.401
Flu-like symptoms	48 (49.0%)	103 (42.6%)	0.335
Annual Influenza vaccine:	47 (48%)	117 (48.3%)	1.00
Sleeping pattern changes	33 (33.7%)	110 (45.5%)	0.053
Regular daily activity changes	36 (36.7%)	109 (45.0%)	0.183
Compliance with personal protective guidelines	87 (88.8%)	211 (87.7%)	0.856
Compliance with recommended gown use	68 (69.4%)	148 (61.2%)	0.172
Compliance with recommended face shield/goggle	43 (54.1%)	122 (50.4%)	0.536
Compliance with recommended gloves use	95 (96.9%)	221 (91.3%)	0.068
Compliance with recommended mask use	88 (89.8%)	217 (89.7%)	0.978
Compliance with hospital ICP policy	93 (94.9%)	229 (94.6.2%)	0.856
Satisfaction with education/training for PPE use	67 (68.4%)	181 (74.8%)	0.229

ICP: Infection control and protection; PPE: Personal protective equipment; SD: Standard deviation.

**Table 2.** Physicians compared with nurses in relation to demographic, health characteristics and compliance/satisfaction with hospital safety measures.

Compared variables	Physicians	Nurses	p-value
Total (n)	113	227	
Age (years), mean (SD)	29.7 (4.32)	31.41 (5.31)	0.000
Experience (years), mean (SD)	4.28 (4.11)	7.6 (4.57)	0.000
Working shift (hours), mean (SD)	17.8 (8.4)	14.1 (6.8)	0.000
Annual influenza vaccine: yes n, (%)	68 (60.2%)	96 (42.3%)	0.003
Flu-like symptoms: yes, n (%)	56 (49.6%)	95 (41.9%)	0.203
Compliance with hospital PP guidelines	109 (96.5%)	213 (93.8%)	0.442
Compliance with hospital ICP policy	102 (90.3%)	196 (86.3%)	0.382
Compliance with recommended gown use	62 (54.9%)	154 (67.8%)	0.023
Compliance with recommended mask	104 (92.0%)	201 (88.5%)	0.351
Compliance with recommended gloves use	102 (90.3%)	214 (94.3%)	0.183
Compliance with recommended face shield/goggle	45 (39.8%)	130 (67.3%)	0.003
Satisfaction with education/training	79 (69.9%)	169 (74.4%)	0.437

ICP: Infection control and protection; SD: Standard deviation.

**Table 3.** Close direct to direct patient's care in relation to healthcare workers’ demographic, occupational and health characteristics.

Compared variables	Close-direct care (n = 260)	Direct care (n = 80)	p-value
Age (years), mean (SD)	30.11 (4.4)	33.3 (6.2)	0.000
Experience (years), mean (SD)	5.92 (4.26)	8.38 (5.50)	0.000
Working shift (hours), mean (SD)	16.5 (7.9)	11.5 (5.0)	0.000
Gender Female Male	77 (29.6%) 183 (70.4%)	21 (26.2%) 59 (73.8%)	0.672
Occupation: Physician Nurse	89 (34.2%) 171 (65.8%)	24 (30.0%) 56 (70.0%)	0.501
Smoking Yes No	56 (21.5%) 204 (78.5%)	23 (29.4%) 56 (70.6%)	0.123
Flu-like symptoms Yes No	122 (46.9%) 138 (53.1%)	29 (36.2%) 51 (63.7%)	0.096
Influenza vaccine Yes No	122(46.9%) 138 (53.1%)	42 (52.5%) 38 (47.5%)	0.441
Sleeping pattern changes Yes No	119 (45.8%) 141 (54.2%)	24 (30.0%) 56 (70.0%)	0.014
Regular daily activities changes Yes No	115 (44.2%) 145 (55.8%)	30 (37.5%) 50 (62.3%)	0.304

SD: Standard deviation.

Close direct care providers were more complaint with personal protective guidelines (96.5%) compared with direct care providers (88.8%) (p = 0.018) and they were more compliant with the hospital infection control/protection policy (90.4%) compared with the direct care providers (78.8%) (p = 0.010). About 68.8% of the close direct care providers were satisfied with the hospital education/training for PEE use compared with those providing direct care (86.2%). Comparison between the close direct care providers and direct care providers concerning compliance with personal protection guideline/ICP policy, recommended PPE use and satisfaction with education/training for PPE is presented in Table 4.

**Table 4.** Close direct to direct patient’s care in relation to healthcare workers compliance and satisfaction with hospital safety measures.

Compared variables	Close-direct care (n = 260)	Direct contact (n = 80)	p-value
Hospital personal-protection guidelines Compliant Noncompliant	251 (96.5%) 9 (3.5%)	71 (88.8%) 9 (11.2%)	0.018
Hospital Infection control/protective policy Compliant Noncompliant	235 (90.4%) 25 (9.6%)	63 (78.8%) 17 (21.2%)	0.010
Recommended gown use Compliant Noncompliant	171 (65.8%) 89 (34.2%)	45 (56.2%) 35 (43.8%)	0.144
Recommended glove use Compliant Noncompliant	239 (91.9%) 21 (8.1%)	77 (96.2%) 3 (3.8%)	0.221
Recommended mask use Compliant Noncompliant	232 (89.2%) 28 (10.8%)	73 (91.2%) 7 (8.8%)	0.68
Recommended face shield/goggle use Compliant Noncompliant	139 (53.5%) 121 (46.5%)	36 (45.0%) 44 (55.0%)	0.202
Education/training for PPE use. Satisfied Unsatisfied	179 (68.8%) 81 (31.2%)	69 (86.2%) 11 (21.2%)	0.002

PPE: Personal protective equipment.

Only three HCWs (nurses) had positive assays for total anti-SARS-CoV-2 immunoglobulin. Two symptomatic nurses with positive RT-PCR test were hospitalized during 23–30 March 2020 and discharged for home isolation after a negative RT-PCR test. Both had a history of contact with confirmed cases outside the hospital (Irbid wedding, family dinner). The third with a positive assay for total anti-SARS-CoV-2 immunoglobulin was an asymptomatic male nurse (direct care provider) with a negative RT-PCR assay upon screening on May 2020.

## Discussion

During the COVID-19 pandemic, the reportedly high risk of HCWs’ hospital-acquired infections intensified fears and concerns [21]. Therefore, international and national health authorities recommended extra safety measures and protective guidelines to assure HCWs’ safety [3,22]. Physical distancing, standardized safety measures, contact/respiratory droplets precautions and early identification/isolation of suspected HCWs were recommended to decrease the risk of transmission in healthcare setup [1,5,23]. As physical distancing is challenging to achieve in a hospital setup [5], COVID-19 hospital care in this study was classified as close direct care and direct care. Several investigators reported an increased risk for HCWs providing close contact care compared with those providing care with physical distancing greater than 1–2 meters [19,24]. The overall prevalence of positive serology assays for HCWs in this study was 0.9%. Of the three HCWs confirmed to have COVID-19, two got the infection from outside hospital contact with confirmed COVID-19 cases. There is accumulative evidence suggesting risk reduction with protected direct care [19,24].

Furthermore, the risk of transmission is high from exposure to undiagnosed asymptomatic patients, coworkers, household members and the community outside hospitals [5,25]. Therefore, it would be difficult to finally judge where the source of the infection was finally from. Although most HCWs in this study were close direct care providers (76.5%), the prevalence of positive immunoglobulin assay was 0.7% compared with 1.2% for direct care providers. This apparent difference cannot be analyzed for significance due to the limited numbers. The low hospital influx of COVID-19 patients, low community transmission during the first pandemic wave in Jordan and overall compliance with personal protective guidelines and ICP policy were responsible for low prevalence in this study. Close direct care providers were more compliant with personal protective guidelines and ICP policy than direct care providers (p < 0.05). A substantial risk reduction was observed for HCWs while providing protected direct care for confirmed patients [26,27]; several other investigators reported a high HCWs risk for transmitted infection due to inadequate training, lack of protective equipment and noncompliance with safety precautions [10,11]. HCWs providing close direct care in this study had to work for long shift hours compared with direct care providers. The hospital policy offers them 10–14 days of home isolation after each close direct care working shift. Even though reports showed an increased risk of HCWs transmitted infection with increasing load of work and long working shifts hours during the early phase of SARS-CoV-2 pandemic [11]. However, nurses had high compliance with the recommended face mask and face shield/eye goggles compared with physicians (p < 0.05), all seropositive assays were found in nurses. Nurses constitute (66.8%) of the study population, and 2/3 of seropositive nurses had an outside hospital contact with confirmed cases, as stated earlier.

One drawback to this study is that emails containing the questionnaire were sent nonanonyms which could carry a biased answer toward a more positive answers in fear of possible accusation and responsibility for breaching rules and regulations.

This study demonstrated a higher influenza vaccination rate among physicians (60.2%) than nurses (42.3%). Al-Mistarehi *et al.* reported that HCWs and those who reported receiving influenza vaccine had higher rates of COVID-19 vaccine acceptance compared with their counterparts (p < 0.001) [28]. There is evidence suggesting reduced mortality in vaccinated elderly Italians [29]. Furthermore, in a mathematical method of viral co-infection, SARS-CoV-2 replication was suppressed easily by many common respiratory viruses [30]. Ragni P *et al.* found no association between influenza vaccinations and outcomes measures for COVID-19 patients; although, the vaccinated individuals were found to have a lower probability of positive RT-PCR testing [31]. In a recent meta-analysis, the estimated prevalence of positive serological assay is 7% among HCWs; nearly half of them are nurses [32].

The prevalence of SARS-CoV-2 infection among HCWs is variable in different studies, depending on the pandemic phase (early vs late) and diagnostic modality (molecular vs serology) in addition to differences in HCWs population studied [32]. Recent reports demonstrated a reduction in hospital-acquired infection among HCWs due to accumulated knowledge, experience, early detection, isolation, understanding of the ICP measures/personal protective guidelines, proper education/training, availability of PPE and compliance [22,33]. Although the optimal and proper PPE is a matter of debate, standardized and high compliance with personal protective guidelines and infection prevention/control policies can dramatically reduce nosocomial transmissions [34]. Despite all these improvements in safety precautions, the risk of SARS-CoV-2 transmission among HCWs is several folds higher than that for the community in general [9]. In this study, HCWs providing close direct care had a higher prevalence of sleeping pattern changes. It was established that a considerable proportion of healthcare personnel reported symptoms of mental health problems, ranging from depression, anxiety, insomnia and stress [22]. Also, a considerable proportion of in-hospital quarantined COVID-19 infected patients reported symptoms of depression, regardless of their COVID-19 infection severity [35]. Data has shown that a fourth of HCWs reported mild anxiety, depression or insomnia [36]. Apart from the constant fear of infecting family members, HCWs also described being stigmatized, as they were assumed to be potential sources of contamination [37].

## Conclusion

The high level of compliance with infection control/protection policy and personal protective guidelines might explain the low prevalence of positive total anti-SARS-CoV-2 immunoglobulin assays among HCWs in a hospital care setup, Further studies needs to be carried out that would help in developing proper protocols for future pandemics in terms of screening, protective measures in addition to surveillance of the level of compliance.

## Future perspective

There is a possibility of having other future outbreaks; that is why early implementation of safety precautions and guidelines in dealing with significant outbreaks needs to be considered. Healthcare sectors need to have sufficient resources of PPE in addition to providing protective protocols that aid in decreasing the rate of spreading the virus among HCWs, as well as having early screening tools to detect and isolate infected employees.

**Summary points**

- COVID-19 pandemic has caused an unpredictable largescale health challenge on the healthcare sectors in general and on healthcare workers (HCWs) in particular.
- COVID-19 outbreaks have caused significant restrictions and have forced to adapt major precautions to decrease the risk of infection among HCWs.
- Physical distancing, standardized safety measures, contact and respiratory droplets precautions, as well as early identification/isolation of suspected HCWs were recommended to decrease the risk of transmission in the healthcare setup.
- Early implementation of the safety measures and compliance to the personal protective guidelines resulted in decreased risk of contracting the virus in the hospital settings.
- Reduction in hospital-acquired infection among HCWs in healthcare facilities can be achieved by accumulated knowledge, experience, early detection, isolation, understanding of the ICP measures/personal protective guidelines, proper education/training, availability of PPE.
